# Who Can I Trust in a Scary World? An Examination of the Objects of Trust, Information Sources and Social Distancing Intention Amid COVID-19

**DOI:** 10.3390/ijerph18105321

**Published:** 2021-05-17

**Authors:** Lu Wei, Tien-Tsung Lee

**Affiliations:** 1 College of Media and International Culture, Zhejiang University, 866 Yuhangtang Rd., Hangzhou 310058, China; drluwei@zju.edu.cn; 2 Department of Communication, Faculty of Social Science, University of Macau, Taipa, Macau, China

**Keywords:** trust, media effects, social distancing, COVID-19

## Abstract

Trust is a central construct of social research. While numerous studies have investigated trust as either a dependent or independent variable, little attention has been paid to its relationship with health-related behaviors in the context of a public health crisis. How trust in different entities influences people’s social distancing intention is therefore an important question that merits academic scrutiny. Moreover, the relationship between trust and social distancing intention cannot be well understood without an account of the information environment. As previous studies have reached a consensus about the limited effects of information exposure on individual outcomes, this research focuses on possible moderating effects. Results show that information exposure, no matter via interpersonal or media sources, has no direct effects on behavioral intention. Rather, risk communication serves as a moderator of the relationship between trust and social distancing intention.

## 1. Introduction

While numerous studies have investigated trust—a key construct in social science research, as a dependent or independent variable—relatively little attention has been paid to its relationship with health-related behaviors in the context of a public health emergency. The outbreak of the coronavirus disease (COVID-19) has posed severe threats to public health and has caused substantial economic and social consequences worldwide. China is the country that first reacted to the pandemic on 31 December 2019, the date when confirmed COVID-19 cases were first reported in Wuhan. During the outbreak, COVID-19 has not only led to morbidity, mortality, and a huge economic cost, but also significantly changed the landscape of social relationships. People have been trying to avoid face-to-face contact with others. Social distancing, including voluntary and mandatory isolation at home, has become a recommended and effective measure to reduce the spread of virus infections in this pandemic [1]. Nevertheless, there have still been a number of individuals who refused to follow social distancing guidelines and kept socializing with others. How trust in different entities would influence people’s social distancing intention is therefore an important question that merits scholarly scrutiny.

This study examines the connection between people’s trust in five types of entities—interpersonal, scientists, the government, social (others in general), and healthcare providers—and how likely they will follow social distancing advisory during a pandemic. The definitions or operationalizations of these key terms will be discussed in the sections below.

The relationship between trust and social distancing intention cannot be well understood without an account of the information environment. For years, conflicting and contradictory results have seemed common among medical studies, such as the preventative effects of beta carotene and aspirin, leaving both experts and consumers often confused about which healthcare-related advice to believe and follow [2,3]. This type of “information noise” highlights the importance of studying the behavioral effects of trust. As pointed out by the World Health Organization [4], the COVID-19 outbreak has been accompanied by a massive “infodemic”, an over-abundance of information—some accurate and some not—that makes it hard for people to find trustworthy sources and reliable guidance when they need it. In such a confusing information environment, people frequently come across information associated with COVID-19 from both interpersonal and media sources. The information about COVID-19 mainly covers confirmed and cured cases, associated health threats, suggested precautions, social support, and socioeconomic consequences of the pandemic [5]. Exposure to this information is expected to influence individuals’ behavioral intention to follow the social distancing policy during the pandemic. As previous studies have reached a consensus about the limited effects of information exposure on individual outcomes, this research focuses on the possible moderating effect. That is, whether the communication of risk via interpersonal and media channels will moderate the relationship between the objects of trust and one’s willingness or intention to social distance during a pandemic.

## 2. Theoretical Background

### 2.1. Trust as a Dependent or Independent Variable

Sztompka argues that we live in an “inter-human space.” Human society is “a network of relations among the people” and “what happens between and among individuals” [6] p. 31. He also points out that human lives are precarious, and people are fragile animals who are exposed to numerous threats. For a society to function well, it needs six moral bonds or values among people, namely trust, loyalty, reciprocity, solidarity, respect, and justice. Among them, trust is the most fundamental one [6].

Trust can be defined as “a bet about the future, contingent actions of others” [6] (p. 35). It can also be understood as the willingness of a trustor “to be vulnerable to the actions of” the trustee based on the expectation that the trustee “will perform a particular action to the trustor, irrespective of the ability to monitor or control that other party (trustee)” [7] (p. 12).

People receive a significant amount of information from the media. The nature and content of such information, and the level of trust in the information itself, can affect their behavior [8,9,10]. As expected, many communication studies that deal with trust focus on audience members’ trust in news media, also known as media credibility. These studies often use the audience’s perceived media bias or favoritism to operationalize media trust. Other variations include media believability, trust/distrust, trustworthiness, cynicism, and skepticism [11,12,13,14,15,16]. Even though these terms are defined or operationalized somewhat differently, their common core is whether or not, or how much, audience members believe the news media (including news media in general, certain news organizations, journalists, news sources, or news reports) can be trusted to report fairly or accurately.

A large portion of studies in this tradition have treated trust in media—or perceived media bias—as a dependent variable. They found that ego-involvement or issue-involvement including one’s position or strength in political ideology (e.g., liberal or conservative) and partisanship (e.g., Republican or Democrat; or support for a group or cause), are often associated with the perception that the media are biased against one’s cause, belief, or group, and therefore not to be trusted [15,16,17]. Other independent variables include social trust (whether or how much other people can be trusted), political trust (trust in political institutions such as the government), and the overall health of the economy. Also, a more positive economic environment seems to produce a higher level of media trust [18,19,20,21]. In addition, the structural diversity of a local community has been found to predict how much its residents trust the news media. Specifically, people who live in a structurally pluralist and politically heterogeneous community (i.e., having more professions or supporting multiple political parties) are less likely to find news media credible [22,23].

While it is helpful to examine the factors predicting media trust, it is natural to wonder about the consequences of media trust or distrust. Logically, a higher level of trust would be associated with more media consumption. Less trust, on the other hand, is usually linked to less media use [15,24]. Fletcher and Park [25] reported that less trust in the news media is associated with a higher level of preference for non-mainstream news sources (e.g., online-only news outlets, social media, and blogs) and a higher level of online news participation (e.g., sharing news stories or commenting). These studies demonstrate that media trust is connected with media use.

Beyond media use, what else does media trust predict? Tsfati and Cohen argued that media trust can moderate media effects (such as audience perceptions of public opinion regarding which political candidate will likely win an election). They also pointed out that “research on the role of media trust in media effects has not been thus far investigated in the context of behavioral effects” [15] (p. 5). One of the exceptions is a study by Eveland and Shah [26], which reported that media distrust might increase political involvement. Ho and colleagues [12] discovered that perceived media bias is negatively but indirectly associated with general political participation. They also reported this bias perception is positively and directly related to issue-specific political participation. Lin [27] found that social media trust positively predicts dual screening use (simultaneously using social media platforms and viewing videos about current affairs on two-screen devices), which subsequently leads to more positive attitudes toward civic engagement, and eventually offline civic engagement behavior. Taken together, these studies suggest that trust can be an effective predictor of various behaviors.

### 2.2. Trust and Health-Related Behavior

A usual consequence of trust is cooperation [15,28]. There is already a sizable amount of research about the effects of trust in the context of health in general or disease prevention in particular. For example, using a series of preventative behaviors as dependent variables (i.e., to clean objects, to wash hands, to use tissues when sneezing, social distancing, and vaccine acceptance) in the context of the 2009 H1N1 influenza pandemic in Italy, Prati, Pietrantoni and Zani [29] found that media trust and trust in the Ministry of Health are predictors of all of the above behaviors. Trust in the institutional response to the outbreak is positively associated with using tissues when sneezing and vaccine acceptance. Trust in medical science is positively associated with cleaning objects and vaccine acceptance. Recall of exposure to a media health campaign is positively connected with only one behavior, namely using tissues when sneezing.

Li and Wang [28] divided trust into three dimensions: benevolence trust (“the degree in which trustees act in trustors’ interests”), competence trust (“the degree to which trustees are capable of meeting trustors’ needs”), and integrity trust (“the degree of trustees’ honesty and reliability”). With the trustee being “online health services” (“a relationship in which individuals already have sought online health information and have some perceptions toward the credibility of health information by observing the consequences of applying the health information”) (p. 6), they found that benevolence trust (path coefficient = 0.23, *p* < 0.01) and competence trust (path coefficient = 0.59, *p* < 0.001) predict intention to seek health information, while the connection between integrity trust and intention is not statistically significant. Ye [30] reported that trust in online health information can lead to “a variety of Internet uses for health” and “self-efficacy belief in taking care of one’s own health.” Self-efficacy is operationalized as overall self-confidence about ones’ ability to take good care of their health. Internet uses for health include among other things buying medicine or supplements online, participating in online support groups, using a website to manage health-related activities, communicating online or via email with health professionals, and looking for healthcare providers. These studies suggest that trust in various objects has positive effects on health-related behaviors or behavioral intentions.

### 2.3. Research about Information Use, Information Seeking, and Trust Related to COVID-19

Understandingly, people must be eager to learn about the dangers of COVID-19. Quite a few researchers have produced useful findings about the predictors, and consequences of information-seeking, and the use of relevant information in the context of this novel virus. For instance, Soroya and colleagues reported that Finnish citizens tend to seek related information from traditional sources including mass media such as print media and the official websites of newspapers, while personal networks and social media are not preferred sources. In addition, they found that exposure to social media can lead to information overload, information anxiety, and information avoidance [31]. Based on a survey of Chinese respondents, Li and Zheng found that online information seeking about COVID-19 is positively predicted by affective responses (such as feeling anxious, concerned, or worried), informational subjective norms (family, friends, and others expect them to seek relevant information), information insufficiency (how much they feel that they know about COVID-19), and prevention intent (how likely they will be to take preventive actions such as wearing a mask and washing hands) [32]. Scopelliti, Pacilli, and Aquino surveyed Italians and discovered that changes in the use of public spaces are positively related to television exposure, the perceptions of preventive measures, and the level of fear [33].

We found few studies related to trust in this context. Among them, Dalog-Way and McComas reflected on three themes of risk communication—trust, tradeoffs, and preparedness, and argued that trust is the most important factor during infectious disease outbreaks. This is a theoretical piece with valuable advice [34]. Based on a survey of Americans, Sailer and colleagues reported that information seeking and science knowledge are positively associated with coronavirus knowledge. Such knowledge helps respondents avoid panic behavior. Also, those lacking science knowledge and knowledge about this virus would follow preventative guidelines if they trust in medicine [35]. In addition, Scopelliti and colleagues found that trust in authorities is positively associated with individual prevention (including washing hands, avoiding touching one’s mouth and nose, and keeping social distance) and social prevention (such as supporting the sanitization of public transport and the control of temperature in public spaces) [33]. Considering the lack of relevant research on trust in the context of the current pandemic, a study about people’s trust in different entities is necessary.

### 2.4. Alterantive Ways of Measuring Media Effects, and the Effects of Interpersonal Communication

Researchers have been fascinated by media effects for many years. Influential theories such as agenda-setting, cultivation, and knowledge gap all suggest that the media have some effects on the audience under some circumstances [8,9,10]. Under various theoretical frameworks, scholars have investigated whether and how much the use of various media would affect knowledge, beliefs, attitudes, or behaviors [36,37]. Individual studies have reported small or moderate sizes of effects. For example, in an investigation into the digital divide, Wei and Hindman [38] measured four interaction effects (Internet × education, television × education, newspaper × education, and radio × education) on political knowledge. The coefficients are all small, with only one being significant (Internet × education, b = 0.16, *p* < 0.05). Using political self-efficacy as the dependent variable, Kushin and Yamamoto [39] discovered that college students’ attention to various media types has no effects (newspaper, TV news, magazine, social media) or limited effects (radio, b = 0.11, *p* < 0.05; traditional Internet sources, b = 0.33, *p* < 0.001) on political participation. Focusing on the amount of time watching TV dramas from South Korea among Vietnamese women, Vu and Lee [40] reported small effects (path coefficients) on the accuracy of perceived reality about South Korea (path coefficient = −0.37, *p* < 0.001), favorability toward South Korea (path coefficient = 0.15, *p* < 0.001), and intention to marry South Korean men (path coefficient = 0.38. *p* < 0.001).

Would it make any difference if the perceived accuracy of news depiction, which is a dimension of media trust, is involved? Fujioka [41] examined the predictors of an audience’s perceived threats of Latinx immigrants. The perceived accuracy of news depiction of these immigrants has a β of 0.18 (*p* < 0.001). In the same study, the perceived accuracy of news coverage also has a small effect on attitudes toward Latinx immigrants (β = −0.12, *p* < 0.10). This example suggests that the attitudinal effects of media trust are limited.

Similar to individual research, meta-analysis studies also found limited media effects. For instance, Valkenburg, Peter, and Walther [37] concluded that meta-analyses typically report small to moderate media effects under *r* = 0.20. Luo, Burley, Moe, and Sui [42] analyzed 43 years of agenda-setting studies and found a moderate mean effect size of 0.487. Such limited effects suggest that media use or attention should probably be viewed and tested as a potential moderator instead of a direct, independent variable.

The Media Practice Model [43,44] posits that media consumption and effects should be understood as a cyclical process with the following four elements: a media user’s identity, media selection, media engagement, and media application (e.g., commenting on or sharing on social media). In other words, it might be less than ideal to test media effects in a linear model along with multiple independent variables at the same time. Instead, a path model might be a better approach.

Even though the Media Practice Model suggests possible mediation effects of media use, media might have a moderating or interaction effect on some occasions. For instance, because knowledge gap and digital divide literature often consider education a key variable [8,45,46,47], the Wei & Hindman [38] study mentioned above tested the interaction effects of education and the use of various types of media. In other words, media use was treated as a moderator on the effects of educational attainment on knowledge gains. Tsfati [48] discovered the interaction effects of media skepticism and web experience in years on the number of websites accessed. Those with more experience (6, 8, and 10 years) demonstrate a negative relationship between skepticism and the number of news sites (a lower level of skepticism is associated with accessing more news sites). Those with less experience (2 years) show a positive association (those with a higher level of skepticism would visit more sites). Holbert and Park [49] pointed out that a moderation variable is a third variable that addresses “when” or “by whom” an effect takes place. In other words, a moderator alters an observed relationship.

The theory of reasoned action (TRA) posits that people make decisions on an intended behavior as the result of available information and rational consideration. One’s behavior intention, if volitional, is the best predictor of actual behavior [50,51,52]. Health information, which is often available through the media, is “critical to promoting healthy lifestyles and preventing unhealthy behaviors” [53] (p. 252). In a virus pandemic, people are likely seeking public health information available to them frequently, and the news media are certainly a major source. Gaziano pointed out that the dissemination of information in an interpersonal context “is often different from mass media diffusion of information” [45] (p. 662). Political communication scholars recognize the importance of interpersonal communication in addition to media effects [54]. In a pandemic, the news media are not the only information source that people will rely on. Individuals will likely seek information or advice from interpersonal sources as well. Specifically, the level or diversity of information sources about the risks of COVID-19 has the potential to alter the relationship between the level of trust (in various objects in the context of handling the pandemic) and one’s intention to engage in a recommended behavior (e.g., social distancing).

The levels of use and trust in information have important implications in health-related behaviors [34,35,53]. The quantity and diversity of information sources—both media and interpersonal—would qualify as a moderator on the connection between trust and intended behavior. Therefore, this study examines the moderation effects of trust (in different entities) and two types of sources of information (media and interpersonal) on the intention to socialize with others amid COVID-19.

**Figure 1:**
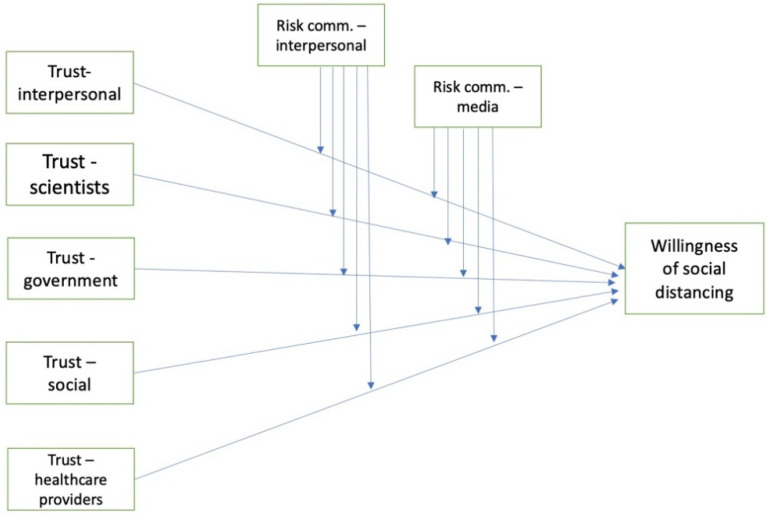
Conceptual model of the relationships between variables to be tested.

Instead of individual hypotheses or research questions, this study asks one overarching research question: which combinations of independent (objects of trust) and moderating (information sources) variables have an effect on the dependent variable (intention to defy social distancing and socialize with others)? Figure 1 is the conceptual model of relevant variables in this study.

## 3. Materials and Methods

### 3.1. Participants and Process

A total of 11,791 people in China were diagnosed with COVID-19 on 31 January 2020, the first time when the number of confirmed cases across mainland China exceeded 10,000. Within a 10-day-period, the confirmed cases surged to 37,626 on 9 February 2020. During this time, apart from the lockdown of Wuhan city, a national social distancing policy was carried out. Most industries and businesses were shut down. People were encouraged to stay home and avoid going to the public space. For those who needed to go out, wearing a mask and keeping social distance were required.

An online survey was conducted between 31 January and 9 February, 2020, an early phase of the COVID-19 outbreak in China. Sojump, a professional online survey company in China, was commissioned to collect data. The company provides a sampling service of 2.6 million registered respondents in China. Within this sampling frame, it first randomly selected 2840 users and then asked them to participate in the online survey through an email invitation. One-thousand-six-hundred-and-fifty-six respondents finished the questionnaire, with a response rate of 58.3%. Despite the under-representativeness of the Chinese population, this sampling strategy is a time-efficient way to explore public reactions and attitudes during the outbreak of COVID-19. Moreover, previous studies have used this sampling strategy to examine major social issues and phenomena in Chinese societies [55,56,57,58,59].

The questionnaire consisted of 179 items in total. Questionnaires with a completion time fewer than 11 min were deleted [60]. Cases that did not pass the attention checks were further excluded. A total of 1568 valid cases were used for data analysis. The sample covered 31 provinces, municipalities, and autonomous regions across mainland China. There were 789 (50.3%) males and 779 (49.7%) females, with a mean age of 31 years old. In terms of household monthly income, 18.1% of the sample (*n* = 284) earned 5000 Chinese Yuan (CNY) or below; 31.1% (*n* = 488) between 5001 and 10,000 CNY; 21.2% (*n* = 332) between 10,001 and 15,000 CNY; 15.8% (*n* = 247) between 15,001 and 20,000 CNY; 12.4% (*n* = 194) between 20,001 and 50,000 CNY; and 1.5% (*n* = 23) above 50,000 CNY. With respect to education, 5.0% of the sample (*n* = 79) were high school graduates or below, 16.1% (*n* = 252) were technical school current students or graduates, 69.1% (*n* = 1083) were current college students or graduates, and 9.8% (*n* = 154) were postgraduates.

### 3.2. Measurements

The dependent variable is the level of one’s willingness to socialize with others during the COVID-19 pandemic. This variable is a combination of five scenarios—including passively accepting invitations and actively initiating gathering—with an α of 0.85. This can be seen as a measure of intended behavior. The independent variables include the trust in five different objects: (1) interpersonal (family, relatives, friends, and strangers in terms of disclosing health conditions and travel histories; α = 0.66); (2) scientists who are working to fight the virus (α = 0.80); the local government of where one lives in their handling of the pandemic (α = 0.85); the generic “others” (“social trust” in terms of how much people can be trusted in general; α = 0.56); and the healthcare providers who treat COVID-19 infections (α = 0.76). These five trustees are included in the analysis because they are directly related to the dealing of this pandemic.

Even though the reliability of social trust is relatively low, we chose to include the trust in amorphous others because the virus itself is invisible to the naked eye and any person can be the source of infection. Prevention of the disease, therefore, depends on the trustworthiness and integrity of others. The implication is whether or not these general “others” would practice social isolation if they knew they have contracted the virus.

Two moderator variables are included in the analysis: (1) risk communication—interpersonal (learning about the risks of this virus from various types of people), and (2) risk communication—media (learning about the risks of this virus from various types of media). The above variables are adopted or adapted from previous studies e.g., [56,57,61,62,63]. Control variables are typical demographic variables in mass and health communication research [28,53], including age, sex, income, and education. Details about the above variables are reported in Table 1. Data were analyzed with Hayes’ PROCESS Macro 3.4.1, Model 1, in SPSS [64]. Bootstrap samples were set at 5000. Continuous variables were mean-centered. Only models with significant moderators are reported.

## 4. Results

We first created a multiple regression model with willingness to social distance as the dependent variable, and the five objects of trust as the independent variables. Control variables are age, sex (male), education, and income. Willingness to defy social distancing guidelines is associated with a younger age (β = −0.09, *p*< 0.01), being male (β = 0.10, *p*< 0.001), having less education (β = −0.11, *p*< 0.001), and not trusting scientists (β = −0.11, *p*< 0.001). The R^2^ of this model is 0.06 (F = 9.26, *p* < 0.001). Trust in other entities are not significant predictors.

Next, a total of 10 moderation models (five independent variables x two moderators) were tested. Only two combinations show significant moderation effects. As demonstrated in Table 2, learning about the risks of COVID-19 from interpersonal sources moderates the effects of interpersonal trust on the intended behavior of social distancing. Those who have a *lower* level of trust in interpersonal sources are *more* likely to report that they will socialize with others (to defy social distancing) (b = −0.07, s.e. = 0.03, *p* < 0.05). That is, the *less* survey respondents trust *others* about the accuracy or honesty of self-reported health conditions or travel histories, the *more* they would go out and socialize. This negative association is counterintuitive, which calls for a closer look at possible interaction effects.

**Figure 2:**
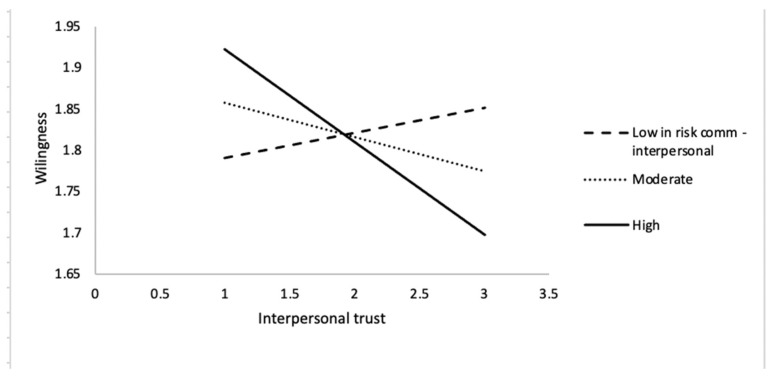
Willingness to socialize with others as a function of interpersonal trust and learning about the risks of COVID-19 from other people.

Risk communication via interpersonal sources has no association with the intended behavior (b = −0.01, s.e. = 0.02, *p* > 0.05). However, the interaction of trust and interpersonal risk communication has negative effects (b = −0.18, s.e. = 0.04, *p* < 0.001; CI [−0.27, −0.10]). In addition, those who are younger (b = −0.01, s.e. = 0.00, *p* < 0.01), male (b = 0.15, s.e. = 0.03, *p* < 0.001), and less educated (b = −0.08, s.e. = 0.02, *p* < 0.001) are more likely to socialize with others amid this virus pandemic. This interaction effect is visualized in Figure 2. Among the respondents with a *lower* level of interpersonal communication about the risks of COVID-19 (meaning they receive *little* information about the risks of COVID-19 from other people), the *more* they trust others, the *more* likely they would be willing to socialize with others. This positive connection is expected.

For those with a moderate and high amount of interpersonal risk communication, the *more* they trust others, the *less* likely they would engage in socializing. In other words, for those who sometimes or often hear about the dangers of COVID-19 from other people, the association between trust and intended behavior is a negative one. That is, the *more* they hear about the risks of COVID-19 from other people, and the *more* they trust other people about self-reported health conditions or travel histories, the *less* likely they would socialize with others. Such associations are no longer counterintuitive.

Data in Table 3 show that social trust (believing that, overall, other people can be trusted) has a *negative* association (b = −0.11, s.e. = 0.03, *p* < 0.001) with one’s willingness to socialize with others during this COVID-19 pandemic. The *more* individuals believe that others are honest and can be trusted, the *less* likely they would choose to socialize with others in the context of COVID-19. This negative connection is counterintuitive.

**Figure 3:**
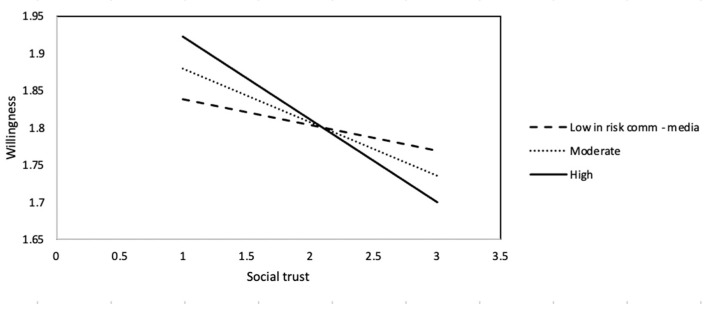
Willingness to socialize with others as a function of social trust and learning about the risks of COVID-19 from the media.

Risk-communication via media itself has no effects on behavioral intention (b = 0.01, s.e. = 0.03, *p* > 0.05). Also, those who are younger (b = −0.01, s.e. = 0.00, *p* < 0.01), male (b = 0.14, s.e. = 0.03, *p* < 0.001), and less educated (b = −0.08, s.e. = 0.02, *p* < 0.001) are more likely to socialize with others despite the pandemic. The interaction between social trust and risk communication via the media has a significant effect on the dependent variable (b = −0.10, s.e. = 0.04, *p* < 0.05, CI [−0.18, −0.02]). The slopes in Figure 3 suggest that among the three groups of people with different levels of learning about the risks of COVID-19 from the media, the *more* they trust other people in general, the *less* likely they would be willing to socialize with others during this pandemic. Higher levels of risk communication via the media seem to intensify the negative association between social trust and willingness to socialize. In other words, the *more* individuals learn about the dangers of COVID-19 from the media, and the *more* they trust others, the *less* likely they would socialize with others during this pandemic.

## 5. Discussion

### 5.1. Significant Findings

The first set of significant interaction effects makes sense. Learning about the dangers of COVID-19 from others alters the effects of interpersonal trust (trusting family, relatives, friends, and strangers about their self-reported health conditions and travel histories) on willingness to socialize amid the pandemic. As shown in Figure 2, if individuals learn little about the risks of the virus from others, the association between interpersonal trust and willingness is a positive one. For those individuals, the *more* they trust others’ self-disclosure, the *more* likely they would choose to go out and meet others. However, if they learn a moderate or high amount of information from others about the risks concerning the virus, even if they trust others’ self-disclosure about health conditions and travel histories, they still tend not to socialize with others. An interpretation is that learning more about such risks from interpersonal sources would overshadow the effects of their trust in others on their behavioral intentions.

As for the second interaction effect, regardless of the amount or diversity of information about the risks of COVID-19 from media sources, social trust (thinking that other people in general can be trusted) has a negative association with one’s willingness to socialize. On the surface, a *higher* level of social trust would lead to a *lower* level of willingness to socialize. That is, the *more* one finds others trustworthy, the *more* they would choose social distancing. Should the relationship not be the opposite—a higher level of social trust would make individuals more willing to socialize with others?

Hearing more about the risks of COVID-19 from the media appears to strengthen the negative effects of social trust on behavioral intention. Therefore, there is a possibility that, under the surface, the real cause of this counterintuitive, negative relationship between social trust and willingness is risk communication via media. In other words, negative information from the media about this virus, regardless of its amount or diversity, would make people believe that other people are subject to the risk of infection and thus strengthen their social distancing intention. If people learn about the dangers of this virus from the media, they are reluctant to socialize with others.

As mentioned earlier, numerous studies, based on such influential theories as agenda-setting, knowledge gap, and cultivation, have consistently discovered media effects [8,9,10]. In our study, which tests the indirect (moderation) effects of risk communication, via the media and interpersonal channels, on the connection between trust and preventive behavior (social distance), we found significant effects in a small number of conditions.

We suspect that an effect of cognitive dissonance is at work [65,66]. This theory refers to psychological discomfort when people struggle between different cognitions. An example used by Festinger is a smoker learning about the dangers of smoking. This smoker can reduce dissonance by either stopping smoking or changing the belief about the harmful effects of smoking [65,66]. The same situation might occur when people debate about whether to go out and socialize with others. They can choose to trust the authorities and the media about the dangers of COVID-19, then decide not to go out and socialize. Or they can choose not to trust the government or other entities regarding the information about the virus, or selectively believe misinformation about COVID-19 via the media and interpersonal communication, then convince themselves that social distancing is not necessary. Future studies about information seeking and other behaviors in a pandemic might want to apply this theory.

### 5.2. Null Findings

In models without interpersonal communication about the risks of COVID-19 as a moderator, with age, sex, income, and education as control variables, a higher level of willingness to socialize is predicted by the following variables involving trust: *less* trust in frontline scientists fighting the virus (b = −0.17, s.e. = 0.03, *p* < 0.001), *less* trust in how the local government handles the pandemic (b = −0.05, s.e. = 0.02, *p* < 0.05), *more* trust in others in general (b = 0.10, s.e. = 0.03, *p* < 0.01), and *less* trust in healthcare providers (b = −0.05, s.e. = 0.02, *p* < 0.05).

In models without significant interaction effects involving the media, and with age, sex, income, and education as controls, a higher level of willingness to socialize with others is predicted by the following: *less* trust in frontline scientists fighting the virus (b = −0.17, s.e. = 0.03, *p* < 0.001), *less* trust in how the local government handles the pandemic (b = −0.05, s.e. = 0.02, *p* < 0.05), *more* trust in others in general (b = 0.10, s.e. = 0.03, *p* < 0.01), and *less* trust in healthcare providers (b = −0.05, s.e. = 0.02, *p* < 0.05). Both sets of analyses produced similar results.

It makes sense that a higher level of social trust (thinking others are trustworthy) is linked to behavioral intention to socialize with others during a virus pandemic. However, why would those with *less* trust in the ability of the scientists, local government, and healthcare providers in terms of handling the pandemic feel more comfortable to socialize with others? One possibility is that they do not take the warnings—about the dangers of the COVID-19 virus—from these entities seriously. Survey respondents likely have the same mentality as some Americans who believe the pandemic is a hoax or at least blown out of proportion. There have been news reports that some Americans ignore social distancing and choose to party instead [67]. However, it should be noted that the mean of the willingness index is 1.80 (s.d. = 0.68) on a 1-to-5-point scale. Therefore, overall people choose social distancing. Nevertheless, the fact that there is a negative relationship between trust in authorities and the willingness to socialize is curious. One possible cause is misinformation about the virus, which is related to the “infodemic” mentioned above [4,68]. This points to a direction for future studies to investigate.

### 5.3. Limitations

A few limitations of this study need to be acknowledged. First, with 69.1% of the survey respondents being current college students or graduates, and 9.8% being postgraduates, our sample is skewed toward those highly educated. This is a common characteristic of online non-probability survey samples [69]. Second, the direction of relationships cannot be established with a survey study. The opposite of the findings can hold true. Third, all the measures of behaviors are self-reported. We cannot verify that such behaviors are actually true instead of socially desirable answers. Fourth, the alphas of some of the measures, such as social trust, are relatively small (<0.70). Despite these limitations, we believe this study has produced useful information to help promote behavior that might prevent the spread of this lethal virus.

## 6. Conclusions

This study has made two contributions. First, it establishes the relationship between trust and intended behavior of social distancing in the context of a public health emergency. Specifically, interpersonal trust and social trust are all negatively associated with the willingness of interacting with others. While this negative connection is counterintuitive, it might reveal the special nature of the pandemic environment. Unlike ordinary daily lives when people tend to socialize with others they trust, in the pandemic situation, people are more likely to keep distance from others when they trust other people who are undergoing a critical risk of infection. Thus, the relationship between trust and behavioral intention is context-dependent. In the risky context, trust may contribute to the acceptance of social distancing policy.

Second, information exposure, no matter via interpersonal or media sources, has no direct effects on behavioral intention. Rather, risk communication serves as a moderator of the relationship between trust and social distancing intention. Particularly, learning more about the risks of the virus, no matter from others or from the media, tend to strengthen the negative effects of trust on behavioral intention. This pattern helps to explain the counterintuitive negative connection between trust and the willingness of interacting with others. The information about the risks of the virus makes people more aware of the risky nature of the pandemic context, so that the more they trust other people about their risky health conditions, the more likely they will keep their social distance from others. Such moderating effects of risk communication deserve more scholarly attention by future studies.

## Figures and Tables

**Table 1:** A summary of dependent, independent, moderator, and control variables.

Variable	Wording	M	S.D.	α
DV: Willingness scale *	(Average index)	1.80	0.68	0.85
	If a relative or friend asked you to eat out, you would accept the invitation because you think you can effectively protect yourself.	1.77	0.91	
	If a relative or friend asked you to be a guest at their home, you would accept the invitation because they have much control over risk in their own home.	1.99	1.02	
	If a relative or friend invited you to a wedding or a funeral, you would accept the invitation because an invitation in this nature is difficult to turn down.	1.75	0.98	
	If an elder invited you out, you would accept the invitation to show respect.	2.03	0.10	
	If a supervisor from work invited you out, you would accept the invitation because it is difficult to say no.	2.09	1.07	
	It is important to maintain a good relationship with relatives during the lunar new year holidays. Therefore, you would initiate visits.	1.60	0.81	
	It is important to socialize with friends during lunar new year holidays. Therefore, you would initiate gatherings.	1.39	0.71	
IV 1: Trust–interpersonal **	(Average index) When you ask the following people about their recent travel histories and health conditions, how much do you trust their answers?	3.63	0.55	0.66
	Family	4.53	0.71	
	Relatives	3.80	0.84	
	Friends	3.85	0.75	
	Strangers	2.35	0.81	
IV 2: Trust–scientists *	(Average scale) How do you rate the scientists who are on the front-line fighting COVID-19?	4.35	0.56	0.80
	They deserve my trust.	4.36	0.64	
	Their professional qualifications are trustworthy.	4.38	0.70	
	They put people’s health as a topic priority.	4.30	0.80	
	I trust them.	4.37	0.71	
IV 3: Trust–government *	(Average scale) How do you rate the performance of the local government of where you live?	3.90	0.70	0.85
	I have confidence in the ability of local government departments in terms of controlling the pandemic.	4.02	0.79	
	Relevant local government departments will fully consider the benefits of the people in the context of the pandemic.	3.85	0.89	
	Relevant local government departments show fairness when dealing with the pandemic.	3.80	0.91	
	Relevant local government departments are transparent when conveying information about the pandemic.	3.80	1.0	
	Overall, relevant local government departments are trustworthy in their handling of the pandemic.	4.03	0.84	
IV 4: Social trust *	(Average scale) How much do you agree with the following statements?	3.34	0.65	0.56
	In daily lives, when I am not careful, people will take advantage of me (reverse-coded).	3.61	0.86	
	Overall, people can be trusted.	3.74	0.80	
	Most of the time, people only look out for themselves (reverse-coded).	2.65	1.00	
IV 5: Trust in healthcare providers *	(Average scale) How much do you agree with the following statements?	3.51	0.75	0.76
	The confirmation rates of COVID-19 in hospitals are low (reverse-coded).	3.91	0.76	
	Hospitals can treat patients effectively because they are over-crowded and have a shortage of supplies (reverse-coded).	2.94	1.11	
	Physicians don’t have enough knowledge about COVID-19 and are prone to misdiagnosis (reverse-coded).	3.93	0.95	
	Hospitals are prone to misdiagnosis because they don’t have enough COVID-19 test kits (reverse-coded).	3.32	1.19	
	Hospitals cannot effectively isolate COVID-19 patients, which will likely cause cross-contamination (reverse-coded).	3.45	1.22	
Moderator 1: Frequency of interpersonal communication about the risk of COVID-19 ***	(Average scale) In the past week, how often did you receive information about COVID-19 from the following sources?	2.71	0.71	
	Relatives (1 = never, 2 = selfdom, 3 = quite a few times, 4 = often, 5 = very often)	4.15	1.01	
	Friends	3.31	1.18	
	Co-workers or classmates	3.0	1.18	
	Healthcare providers	1.77	1.04	
	Workers in community	2.13	1.04	
	Other people	1.92	0.94	
Moderator 2: Frequency of accessing information about the risk of COVID-19 ***	(Average scale) In the past week, how often did you receive information about COVID-19 from the following sources?	3.30	0.60	
	Newspapers or magazines (print)	1.97	1.21	
	Television	3.92	1.12	
	Radio	2.57	1.31	
	Texts messages on mobile phone	3.61	1.14	
	Online information portal sites (e.g., NetEase, Tencent)	3.97	1.09	
	Social media (e.g., Weibo, WeChat)	4.44	0.79	
	Apps or websites of news organizations (e.g., People’s Daily, Toutiao)	4.01	1.14	
	Video apps (e.g., Douyin/Tiktok, Pearvideo)	3.38	1.29	
	Q&A sites (e.g., Zhihu)	2.74	1.22	
	Search engines (e.g., Baidu)	3.31	1.16	
	Learning sites or apps (e.g., Xuexi)	2.48	1.30	
Control 1:	Age (range = 16 to 67)	31.02	9.0	
Control 2:	Sex (M = 50.3%; F = 49.7%); Dummy-coded male			
Control 3:	Education (1–9 scale; 1 = no schooling; 6 = professional college; 7 = bachelor’s degree; 9 = doctorate)	6.76	0.92	
Control 4:	Monthly income (1–10 scale; 1 = no income; 6 = 8001 to 10,000; 10 = 50,001 and above CNY or RMB)	6.29	1.96	

Notes. * 1 = totally disagree; 2 = disagree; 3 = neither disagree nor agree; 4 = agree; 5 = totally agree. ** 1 = totally trust; 7 = totally distrust. *** 1 = none; 2 = selfdom; 3 = several times; 4 = often; 5 = very frequently.

**Table 2:** A summary of the predictors of willingness to socialize with others; IV (X) = Interpersonal trust; Moderator (W) = Risk communication—interpersonal.

Variable	Co-efficient	s.e.	t	LLCI	ULCI
Constant	2.55 ***	0.15	17.44	2.26	2.83
X = Interpersonal trust	−0.07 *	0.03	−2.39	−0.14	−0.01
W = Risk communication—interpersonal	−0.01	0.02	−0.30	−0.05	0.04
XW–interaction	−0.18 ***	0.04	−4.29	−0.27	−0.10
Age	−0.01 **	0.00	−3.09	−0.01	−0.00
Gender	0.15 ***	0.03	4.55	0.09	0.22
Income	−0.01	0.01	−0.93	−0.03	0.01
Education	−0.08 ***	0.02	−4.16	−0.12	−0.04

R^2^ = 0.05, F(7, 1560) = 11.03 *** (the F-statistic of 11.03 is significant at the *p* < 0.001 level); * *p* < 0.05, ** *p* < 0.01, *** *p* < 0.001; LLCI = lower level of confidence interval, ULCI = upper level of confidence interval.

**Table 3:** A summary of the predictors of willingness to socialize with others; IV (X) = Social trust; Moderator (W) = Risk communication—media.

Variable	Coefficient	s.e.	t	LLCI	ULCI
Constant	2.52 ***	0.15	17.43	2.24	2.81
X = Social trust	−0.11 ***	0.03	−4.28	−0.16	−0.06
W = Risk communication—media	0.01	0.03	0.24	−0.05	0.06
XW—interaction	−0.10 *	0.04	−2.35	−0.18	−0.02
Age	−0.01 **	0.00	−2.88	−0.01	−0.00
Gender	0.14 ***	0.03	4.16	0.07	0.21
Income	−0.01	0.01	−0.85	−0.03	0.01
Education	−0.08 ***	0.02	−4.15	−0.12	−0.04

R^2^ = 0.05, F(7, 1560) = 11.15 *** (the F-statistic of 11.03 is significant at the *p* < 0.001 level); * *p* < 0.05, ** *p* < 0.01, *** *p* < 0.001; LLCI = lower level of confidence interval, ULCI = upper level of confidence interval.

## Data Availability

The data were retrieved from the website of National Health Commission of the People’s Republic of China (http://www.nhc.gov.cn/xcs/yqtb/list_gzbd.shtml) (accessed on 30 March 2020).
